# Assessment of Segmental Postural Control During Reaching in Typically Developing Children Using a Single Inertial Measurement Unit

**DOI:** 10.3390/jcm15135113

**Published:** 2026-07-01

**Authors:** Ashley Schilling, David Levine, Jim Richards

**Affiliations:** 1 Department of Physical Therapy, University of Tennessee at Chattanooga, Chattanooga, TN 37403, USA; david-levine@utc.edu; 2 Allied Health Research Unit, Brook Building, University of Lancashire, Preston PR1 2HE, UK; jrichards@lancashire.ac.uk

**Keywords:** postural control, inertial measurement unit (IMU), Segmental Assessment of Trunk Control (SATCo), sit and reach, spinal segment support, pediatrics

## Abstract

**Background:** Clinicians working with children with neuromotor impairments require sensitive measures to assess postural control and evaluate interventions. This study explored the sensitivity of a single Inertial Measurement Unit (IMU) to changes in postural control during reaching in sitting with clinician support at different segmental levels: upper thoracic, lower thoracic, lower lumbar, and no support. The effect of pelvic-stabilizing straps was examined. **Methods:** A single Delsys Trigno IMU sensor attached over the mid-thoracic spine recorded acceleration and angular velocity data during a reaching task in sitting in ten typically developing children. **Results:** Comparisons of the support levels showed a significantly lower range of accelerations in the medial–lateral and anterior–posterior directions when support was provided at the upper thoracic level compared to support at the lower lumbar level. The range of angular velocity in the sagittal and coronal planes showed progressively lower values as the level of support moved cranially. Pelvic stability straps allowed for a significantly greater range of acceleration values in all directions and a greater range of angular velocities in the sagittal and transverse planes. **Conclusions:** These exploratory findings suggest that IMUs may have clinical utility in postural control assessment and evaluating the effects of intervention in children with neuromotor impairment.

## 1. Introduction

Adequate postural control is foundational to functional independence in children, supporting the capacity to perform activities of daily living across developmental stages [1]. For clinicians working with children with neuromotor impairments, sensitive assessments of postural control deficits are essential to understand their impact on function and to guide targeted interventions [2,3,4,5]. Currently available tools for assessing upright sitting trunk control in children rely on observer-rated ordinal scoring and are unable to capture kinematic changes driving clinical judgements. Some of these assessments also require independent sitting, which excludes children with moderate-to-severe motor impairments, and most do not account for the segmental nature of trunk control [6,7,8].

The Segmental Assessment of Trunk Control (SATCo) assessment was developed as a clinically accessible tool to assess postural control in infants and children with neuromotor developmental impairments [3]. The Seated Postural and Reaching Control (SP&R-co) tool built upon this framework to allow a more specific assessment of postural control during manipulation tasks and reaching in sitting [9]. For both assessments, a clinician provides manual support at discrete segmental levels of the trunk, stabilizing all segments below the point of support. Segments above this external support remain under the child’s control, thereby allowing for identification of the highest segmental level at which control is lost when challenged. To ensure that postural responses at higher trunk segments are not impacted by pelvic movement or malalignment during testing, both testing protocols use a pelvic strapping system to maintain a neutral vertical pelvic position [3]. Objectivity and feasibility limitations of these frameworks include ordinal scoring reliant on clinician observations, the need for two examiners, and a recommendation for video recording [3,9]. To date, limited quantitative data exist to support the observations made during a SATCo assessment, thus limiting the selection and evaluation of interventions targeting specific segmental levels of trunk control to a less objective and less informative measurement framework [10]. To prevent the use of “black box” interventions and to address outcome measures becoming a primary barrier to identifying predictors of treatment response, clinicians should rely on measurements that help to answer the question of how an intervention works, not simply if it does or does not work [11,12]. For example, the SATCo tells a clinician whether a child has control at a given level but not how much movement is occurring, in which direction, and with what speed.

When visually assessing movement quality and control, clinicians are known to demonstrate poor interrater reliability, as well as limited ability to recall pre- and post-treatment status, both of which make it difficult to determine whether clinically meaningful changes have occurred [13]. Richards et al. demonstrated that segment angular velocity obtained from video analysis could provide a sensitive objective measure of movement quality to track changes over time [14]. However, instrumentation capable of quantifying movement in this way remained expensive and time-consuming, creating a significant barrier to its use in the assessment of interventions within clinical practice [8,10].

The emergence of wearable inertial measurement units (IMUs) in human movement analysis in around 2010 addressed this access barrier. IMUs integrate accelerometers and gyroscopes in a compact, portable, and user-friendly form. The use of IMUs to quantify movement has become increasingly accepted in clinical research [15,16,17,18,19], including in the assessment of trunk angular velocity, which has been validated as a convenient substitute for force-plate-based postural stability outcomes [20]. Given these advancements, a single IMU affixed at the mid-thoracic spine, used within a segmental postural control framework, has the potential to provide objective measurements of trunk angular velocity and linear acceleration in the sagittal, coronal, and transverse planes. This approach could provide clinically accessible, objective kinematic measures with a level of sensitivity not achievable through observational assessments. Consistent with Phase 1 of the framework for developing and evaluating complex interventions, as described by Skivington et al., this study addresses key uncertainties, a prerequisite to future feasibility and effectiveness investigations of IMU-based postural control assessment [21]. Therefore, the aim of this study was to explore if direct measures of linear acceleration and angular velocity from a single IMU were sensitive to changes in postural control with clinician support at different segmental levels of the trunk during a basic reaching task in sitting, as well as to examine the effect of pelvic-stabilizing straps and differences between reach directions within a typically developing pediatric population. In addition, this study aimed to provide a preliminary data set which may be useful for comparison when considering children with motor impairments.

## 2. Methods

This was a repeated-measures observational study to determine the effect of different levels of clinician support of the trunk and the use of pelvic-stabilizing straps using a single IMU sensor. A convenience sample of typically developing children aged 4–6 years, with no significant medical history impacting the development of postural control, was recruited through word of mouth among university faculty, staff, and students at the University of Tennessee at Chattanooga. Inclusion criteria required that participants demonstrated typical development with no significant medical history. Exclusion criteria consisted of any neurological, musculoskeletal, or other medical condition that would impact or delay typical motor development. Eligibility was confirmed via parent report, and prior to enrollment, parents/guardians were asked to verify that their child met all inclusion criteria and had no conditions meeting the exclusion criteria. All parents/guardians gave consent and all children gave assent before data collection, and the study was approved by the IRB of the University of Tennessee at Chattanooga (Ref: 24-007) and conducted in accordance with the Declaration of Helsinki.

**Figure 1:**
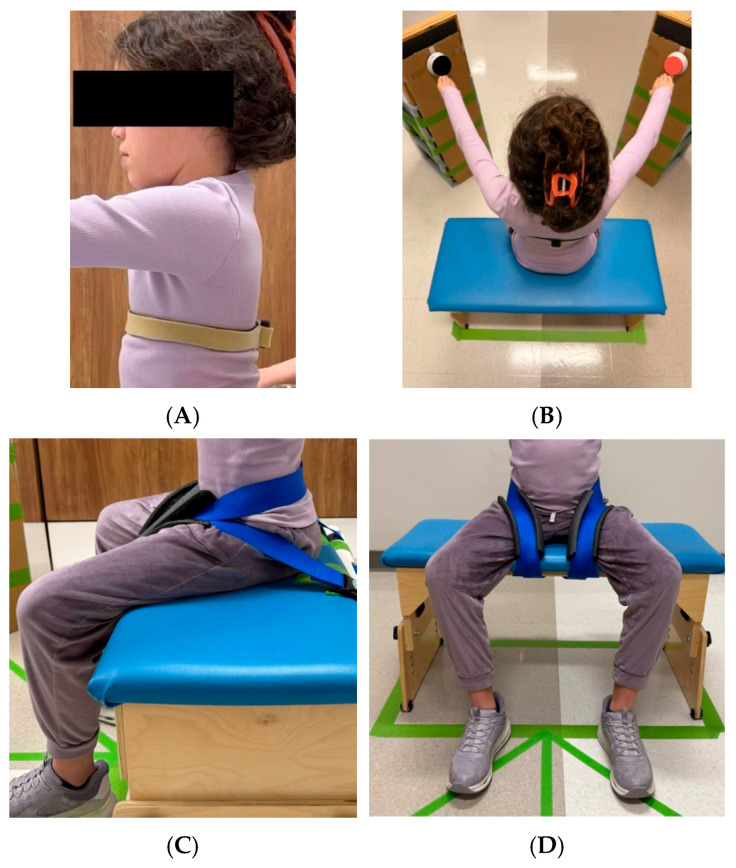
(**A**) Lateral view to show sensor location at the mid-thoracic spine (T7–T9) using a strap with a pocket for the sensor. (**B**) Overhead view of reaching task set-up, with large buttons placed 10 cm further than arm length at an angle of 45 degrees to the right and left of sagittal plane and level with the child’s arm in 90 deg of shoulder flexion and 45 degrees of shoulder abduction to ensure a standardized active reach was performed. (**C**) Anterior and (**D**) lateral view of pelvic straps used to secure the pelvis in a neutral vertical posture, as described in the SATCo protocol.

**Figure 2:**
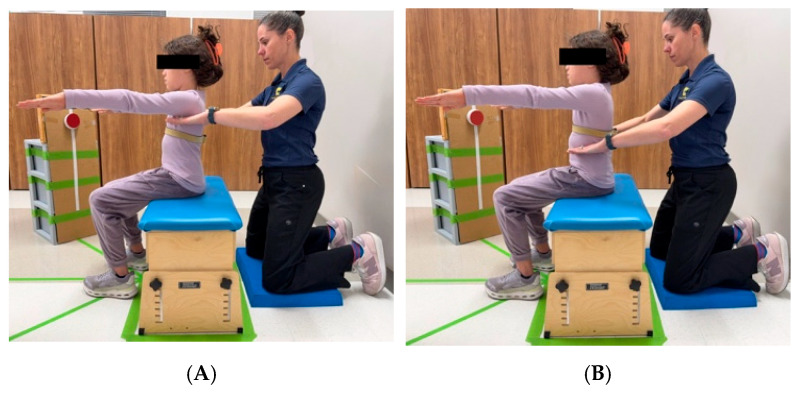

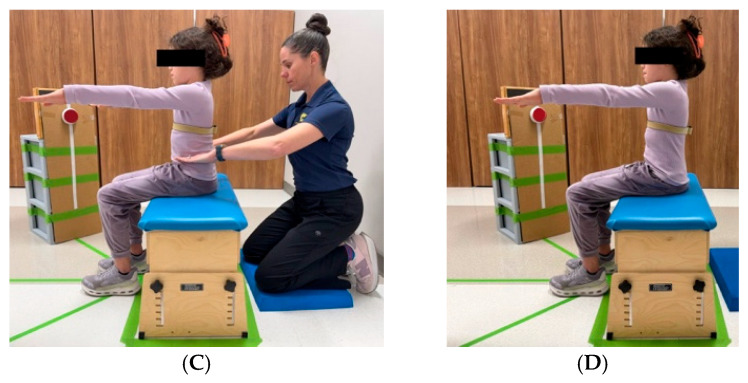
Provision of different levels of support using the SATCo protocol was conducted with the therapist’s hands encircling the segment directly beneath the segment being tested. Support should be horizontal with the aim of stabilizing below the tested segment. Tested segments included: (**A**) upper thoracic (axillae), (**B**) lower thoracic (lower ribs), (**C**) lower lumbar (pelvis), and (**D**) no support.

Scripted instructions were used for all sessions, and all testing was conducted in the same dedicated laboratory space with standardized equipment, lighting, and minimal environmental distractions. A single Delsys Trigno IM sensor (Delsys Inc., Boston, MA, USA) was attached over the mid-thoracic spine (T7-T9) using a strap with a pocket for the sensor (Figure 1), and acceleration and angular velocity data were recorded in three directions and planes at 148 Hz, which was used to explore changes during a basic reaching task to the left and right. The tasks involved an upper-extremity start position of the hand raised to shoulder height with the elbow extended. The participants were asked to reach and press one of two large buttons placed at an angle of 45 degrees to the right and left of the sagittal plane and then return to the start position (Figure 1). The buttons were secured to a stable surface and adjusted to each participant’s shoulder height at a customized distance requiring trunk movement in the direction of the reach, without altering their base of support. The researchers provided demonstrations and allowed the participants to practice the task prior to data collection. Levels of clinician support were based on the SATCo, an observational clinical tool used to assess a child’s ability to maintain postural control with external support provided at different levels on the trunk [3]. Participants completed three reaching trials in each direction, right and left, with support provided across four conditions in a fixed order following the SATCo protocol: upper thoracic (UT), lower thoracic (LT), lower lumbar (LL), and no support or full trunk movement (FT) (Figure 2). A second set of three reaching trials was completed in identical fashion with the application of pelvic-stabilizing straps (Figure 1). A rest break preceded pelvic strap application and participant-requested breaks were permitted to minimize fatigue.

Gyroscope (angular velocity) and accelerometer (linear acceleration) data were exported from Delsys Discover (Delsys Inc., Boston, MA, USA) software to c3d format and imported into Visual3D version 2025.08.1 (HAS Motion, Kingston, ON, Canada). Data were filtered using a 4th order low-pass Butterworth filter with a cutoff frequency of 10 Hz. The onset and end of the movements for each reach repetition were identified when the mid-thoracic spine angular velocity reached a threshold of 5 deg/s. The range of angular velocity in the sagittal, coronal, and transverse planes and range of linear accelerations in the mediolateral and anteroposterior directions were averaged from the three repetitions.

### Statistics

All data were examined and found to be normally distributed using Shapiro–Wilk tests. The main effects of levels of support, the use of straps, and the effect of side were explored using 4 × 2 × 2 repeated-measures ANOVA tests. Least significant difference (LSD) pairwise comparisons were then used to investigate any main effects where significant differences were seen, and effect sizes were calculated using partial eta squared. If the variances of the differences between all combinations were not equal (i.e., Sphericity was violated), Greenhouse–Geisser corrections were applied. All statistics were performed using SPSS v31 (IBM, Armonk, NY, USA).

## 3. Results

This exploratory study recruited ten typically developing children, six males and four females, aged 4 to 6 years old. Children with known neurological, musculoskeletal, or other medical conditions which would impact or delay motor development were excluded. No specific power calculation could be performed as comparable data does not currently exist.

No interaction effects were seen between levels of support, the use of straps, and the effect of side. Significant main effects were seen for the levels of support for all ranges of acceleration and angular velocity measures. For straps versus no straps, significant main effects were seen in the medial–lateral and anterior–posterior ranges of acceleration and in the sagittal and transverse plane ranges of angular velocity, and a significant main effect was seen for side in the coronal plane angular velocity (Table 1).

**Figure 3:**
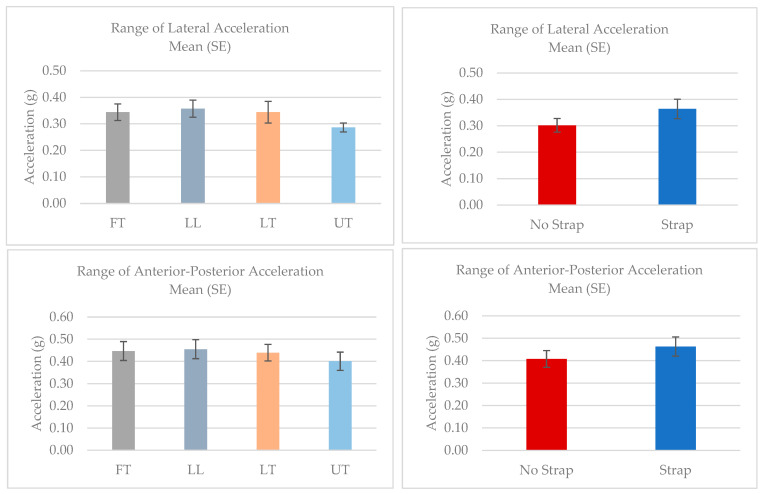
Bar charts showing means and standard errors (SEs) for significant main effects for range of acceleration for the different levels of support; upper thoracic (UT), lower thoracic (LT), lower lumbar (LL), and no support/full trunk movement (FT); and the effect of pelvic stability straps (no strap and straps).

Post hoc pairwise comparisons for the levels of support showed a significantly lower range of accelerations in the medial–lateral and anterior–posterior directions when support was provided at the upper thoracic level compared to support at the lower lumbar level (20% and 12%), respectively, and when support was provided at the upper thoracic level compared to no support (17% and 10%) for the medial–lateral and anterior–posterior directions, respectively (Table 2, Figure 3).

**Figure 4:**
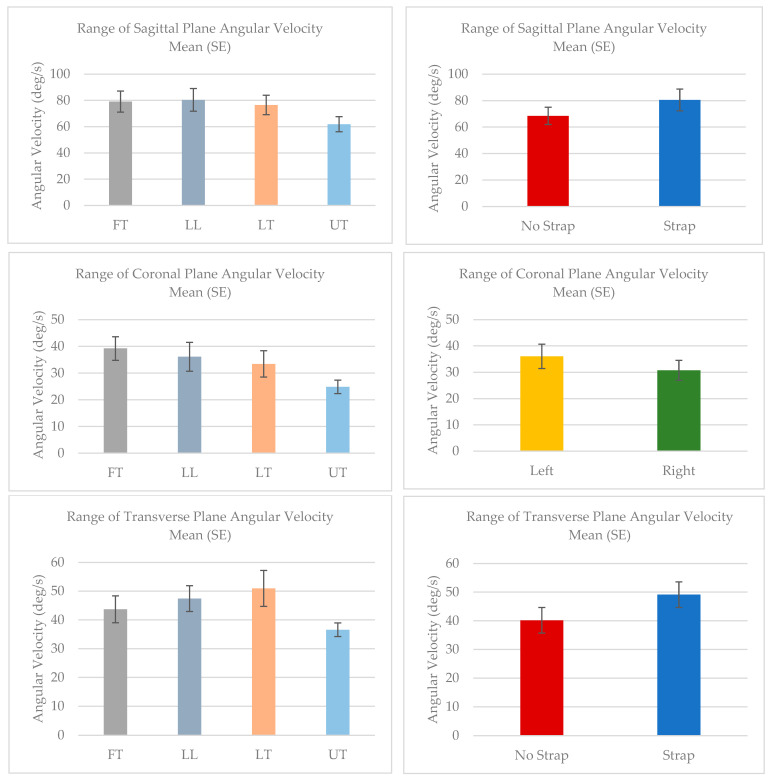
Bar charts showing means and standard errors (SEs) for main effects for range of angular velocity for the different levels of support; upper thoracic (UT), lower thoracic (LT), lower lumbar (LL) and no support/full trunk movement (FT); and the effect of pelvic stability straps (no strap and straps).

Post hoc pairwise comparisons for the levels of support for the range of angular velocity in the sagittal and coronal planes showed progressively and significantly lower values as the level of support was moved cranially. Specifically for the sagittal plane range of angular velocity, upper thoracic support showed 22% lower values than no support, 19% lower values than lower lumbar support, and 23% lower values than lower thoracic support. For the range of angular velocity in the coronal plane, lower thoracic support showed 15% lower values than no support, upper thoracic support showed 37% lower values than no support, lower thoracic support showed 7% lower values than lower lumbar support, upper thoracic support showed 31% lower values than lower lumbar support, and upper thoracic support showed 26% lower values than lower thoracic support. For the range of angular velocity in the transverse plane, upper thoracic support showed 23% lower values than lower lumbar support and 28% lower values than lower thoracic support; however, conversely, lower thoracic support showed 17% greater values than no support (Table 2, Figure 4).

When pelvic stability straps were applied, a significantly greater range of acceleration values was seen in all directions, and a greater range of angular velocities was seen in the sagittal and transverse planes. Specifically, medial–lateral and anterior–posterior accelerations showed 20% and 13% greater values, respectively, with straps, with the sagittal and transverse plane ranges of angular velocities showing 17% and 22% greater values, respectively. Left reaching showed a significantly greater angular velocity range in the coronal plane than right reaching, with a 15% difference between sides (Table 2, Figure 3 and Figure 4).

## 4. Discussion

This exploratory study demonstrated the sensitivity of a single IMU placed at the mid-thoracic spine to detect differences in trunk acceleration and angular velocity across discrete segmental levels of clinician support during a seated reaching task in typically developing children aged 4–6 years. Currently available clinical tools, such as SATCo and SP&R-co, rely on observer-rated ordinal scoring that cannot capture segment kinematic data; the present findings demonstrate that an IMU used within a segmental support framework provides objective, quantitative data sensitive to changes at each support level, addressing this fundamental measurement limitation.

The ability to accurately identify postural control deficits, select targeted interventions, and consistently document change is contingent on measurement sensitivity. Observer-rated ordinal scoring limits clinicians to broad categorical judgements about whether control is adequate or inadequate at a given level, providing no quantitative basis for distinguishing subtle differences in control quality, tracking gradual improvement, or demonstrating treatment-related change [10,11,13]. The present findings suggest that a single mid-thoracic IMU used within a segmental support framework can address this gap by providing quantitative, sensitive data to determine the effects of the different levels of trunk support, which has direct relevance to clinical decision-making.

When support was provided at progressively higher levels of the trunk, the range of angular velocity measured at the mid-thoracic spine decreased in the sagittal and coronal planes, with the greatest reductions seen at the upper thoracic level. These decreasing values may indicate that as more of the trunk was externally stabilized, less active postural control was required of the child. This is a pattern that is both clinically intuitive and consistent with the segmental framework of the SATCo protocol [3]. Importantly, the IMU detected meaningful differences between each level of support, providing objective, quantitative discrimination that existing observational tools are unable to achieve. Given the sensitivity of a single IMU to detect level-dependent changes in trunk kinematics during reaching in children without trunk control deficits, the use of an IMU offers a potential method to detect and quantify kinematic differences in children with trunk control deficits and may also be able to capture more nuanced postural control disruptions in children whose trunk control deficits are less visible [22].

The range of angular velocity in the transverse plane showed a different pattern from the sagittal and coronal planes, increasing with increased trunk external stabilization, with an exception at the upper thoracic level. This finding may reflect the anatomical structure of the spine, where axial rotation range of motion is greatest in the upper and mid-thoracic segments and decreases progressively toward the lower thoracic spine. This suggests that when external support was applied at the upper thoracic level, the segment with the greatest inherent rotational capacity was mechanically constrained, producing a reduction in transverse plane angular velocity independent of active postural control.

Both the SATCo and SP&R-co protocols recommend pelvic strapping to provide a stable base of support and prevent pelvic movement from confounding postural responses at more proximal segments [3,9]. The finding that acceleration and angular velocity ranges were significantly greater across all support levels when pelvic-stabilizing straps were used suggests that stabilizing the pelvis passively using straps may have increased the children’s confidence in their base of support or offered proprioceptive input. This finding may have important implications for clinical use of pelvic strapping in both assessment and intervention contexts and warrants further investigation.

To our knowledge, this is the first study to examine IMU sensitivity to changes in segmental trunk postural control in children, and these data provide reference values for typically developing children aged 4–6 years that may serve as a comparator for future studies in children with atypical postural control. No known studies had previously used IMUs to examine the effect of segmental trunk support level on postural control during reaching in children, and limited quantitative data existed to inform clinical decision-making regarding interventions targeting specific segmental levels of trunk control. The current findings begin to address this gap, suggesting that IMU-derived measures of angular velocity and acceleration range are sensitive to detecting segmental changes in trunk control and may be capable of detecting clinically meaningful change following interventions. This line of investigation is supported by a recent call for the use of accelerometry-based outcomes in a large-scale systematic review investigating predictors of therapeutic outcomes [12], and highlights the need for new assessments of segmental trunk control which are appropriate for use in younger children and those with cognitive or attentional impairments. This phase 1 study intentionally establishes proof-of-concept sensitivity; other questions concerning clinical applications, workflow, equipment requirements, and cost-effectiveness will be addressed in subsequent feasibility and implementation phases [21].

### Limitations

The small sample size and convenience sampling method limit the generalizability of these findings. No standardization of time of day for testing was used to make scheduling easier for participating families, but the laboratory environment was the same for each participant. Future studies should include larger, more diverse samples and extend this methodology to children with neuromotor diagnoses to establish clinical utility in the assessment of segmental trunk control in atypically developing children with known trunk control deficits.

## 5. Conclusions

This exploratory study provides preliminary evidence that a single IMU placed at the mid-thoracic spine may be sensitive to changes in segmental trunk postural control during seated reaching tasks in typically developing children aged 4–6 years, providing objective, kinematic data that no currently available observational clinical tool can achieve. Angular velocity, a proxy measure of movement quality and control, decreased systematically as trunk support was moved cranially, suggesting that the IMU may be capable of quantitatively discriminating between the segmental levels defined in the SATCo protocol. The unexpected finding that acceleration ranges were greater with pelvic-stabilizing straps may suggest that a secure base of support increases children’s confidence to move freely during reaching. To our knowledge, this is the first study to explore IMU sensitivity to assess segmental trunk postural control in children, and the preliminary reference data generated for this age group may provide a foundation for future research examining IMU-based postural control assessment and offer potential clinical utility in the assessment of children with neuromotor impairments.

### Clinical Relevance

This study demonstrated that a single mid-thoracic IMU can detect postural changes during functional reaching tasks in children. These results provide quantitative evidence for the effect of different levels of clinician support when assessing sitting postural control during reaching. This method may have clinical utility to track progress in children with neurological disorders and the effect of interventions.

## Figures and Tables

**Table 1:** Means and standard deviations (sd) and main effects with effect sizes (pn2) for 3-factor ANOVA exploring levels, side, and effect of straps. Bold indicates significant main effects.

		Mean (sd)				*p* Value (ƞ^2^p)		
		No Support (FT)	Lower Lumbar (LL)	Lower Thoracic (LT)	Upper Thoracic (UT)	Levels	L v R	Straps v No Straps
Range of Linear Acceleration								
Medial–Lateral	L No Straps	0.308 (0.112)	0.350 (0.122)	0.312 (0.116)	0.292 (0.123)	**0.005 (0.38)**	0.465 (0.06)	**0.046 (0.37)**
	L Straps	0.398 (0.096)	0.374 (0.113)	0.388 (0.193)	0.324 (0.071)			
	R No Straps	0.313 (0.141)	0.316 (0.105)	0.303 (0.094)	0.221 (0.054)			
	R Straps	0.358 (0.141)	0.389 (0.162)	0.374 (0.196)	0.309 (0.073)			
Anterior–Posterior	L No Straps	0.422 (0.148)	0.420 (0.160)	0.392 (0.108)	0.375 (0.107)	**0.036 (0.27)**	0.546 (0.04)	**0.017 (0.48)**
	L Straps	0.444 (0.140)	0.450 (0.191)	0.471 (0.204)	0.437 (0.167)			
	R No Straps	0.451 (0.192)	0.458 (0.117)	0.402 (0.135)	0.347 (0.121)			
	R Straps	0.470 (0.130)	0.491 (0.152)	0.492 (0.142)	0.445 (0.173)			
Range of Angular Velocities								
Sagittal	L No Straps	77.6 (28.9)	80.9 (32.4)	65.7 (21.5)	57.0 (18.4)	**<0.001 (0.45)**	0.703 (0.02)	**0.034 (0.41)**
	L Straps	81.7 (30.7)	81.8 (38.4)	87.4 (39.8)	73.7 (31.4)			
	R No Straps	78.5 (40.7)	73.6 (20.3)	68.8 (31.3)	45.6 (13.4)			
	R Straps	78.6 (24.0)	85.2 (31.3)	84.3 (28.2)	71.2 (31.5)			
Coronal	L No Straps	44.5 (17.5)	42.3 (29.2)	35.5 (16.3)	27.9 (9.7)	**<0.001 (0.55)**	**0.017 (0.48)**	0.71 (0.016)
	L Straps	40.0 (16.9)	33.2 (14.4)	39.9 (26.2)	25.1 (11.6)			
	R No Straps	35.7 (12.3)	37.7 (13.7)	27.8 (12.6)	19.2 (6.2)			
	R Straps	36.7 (19.9)	31.3 (16.4)	30.6 (17.1)	27.3 (9.7)			
Transverse	L No Straps	41.9 (24.6)	48.0 (21.9)	46.8 (22.2)	34.7 (16.4)	**0.001 (0.44)**	0.268 (0.13)	**0.018 (0.48)**
	L Straps	53.1 (19.1)	52.8 (14.0)	60.7 (35.6)	43.6 (10.8)			
	R No Straps	37.5 (16.8)	41.9 (16.2)	44.2 (17.3)	26.4 (7.4)			
	R Straps	42.3 (15.0)	47.0 (18.8)	51.9 (20.3)	41.7 (12.1)			

**Table 2:** Least significant difference post hoc pairwise comparisons where significant main effects were seen. Bold indicates significant differences.

Parameter	Pairwise Comparisons	Mean Diff	%	*p* Value	95% Confidence Interval for Differences	
					Lower	Upper
Range of Linear Acceleration						
Medial–Lateral	No support v Lower Lumbar	−0.014	−4.1	0.205	−0.036	0.009
	No support v Lower Thoracic	0.000	0.00	0.992	−0.041	0.04
	No support v Upper Thoracic	0.058	16.9	**0.010**	0.017	0.098
	Lower Lumbar v Lower Thoracic	0.013	3.6	0.352	−0.017	0.044
	Lower Lumbar v Upper Thoracic	0.071	19.9	**0.008**	0.024	0.118
	Lower Thoracic v Upper Thoracic	0.058	16.8	0.080	−0.008	0.124
Anterior–Posterior	No support v Lower Lumbar	−0.008	−1.8	0.482	−0.035	0.018
	No support v Lower Thoracic	0.007	1.6	0.681	−0.032	0.046
	No support v Upper Thoracic	0.045	10.1	**0.021**	0.009	0.082
	Lower Lumbar v Lower Thoracic	0.016	3.5	0.317	−0.018	0.049
	Lower Lumbar v Upper Thoracic	0.054	11.9	**0.038**	0.004	0.104
	Lower Thoracic v Upper Thoracic	0.038	8.6	0.172	−0.02	0.096
Medial–Lateral	No Strap v Strap	−0.062	−20.5	**0.046**	−0.124	−0.001
Anterior–Posterior	No Strap v Strap	−0.054	−13.2	**0.017**	−0.096	−0.012
Range of Angular Velocities						
Sagittal	No support v Lower Lumbar	−1.27	−1.6	0.754	−10.13	7.60
	No support v Lower Thoracic	2.58	3.3	0.489	−5.52	10.68
	No support v Upper Thoracic	17.22	21.8	**0.007**	5.95	28.49
	Lower Lumbar v Lower Thoracic	3.85	4.8	0.387	−5.72	13.42
	Lower Lumbar v Upper Thoracic	18.49	23.0	**0.002**	8.41	28.57
	Lower Thoracic v Upper Thoracic	14.64	**19.1**	**0.020**	2.96	26.31
Coronal	No support v Lower Lumbar	3.11	7.9	0.208	−2.08	8.29
	No support v Lower Thoracic	5.79	14.7	**0.008**	1.92	9.66
	No support v Upper Thoracic	14.37	36.6	**0.002**	6.89	21.85
	Lower Lumbar v Lower Thoracic	2.68	7.4	**0.034**	0.26	5.11
	Lower Lumbar v Upper Thoracic	11.26	31.2	**0.010**	3.44	19.08
	Lower Thoracic v Upper Thoracic	8.58	25.6	**0.024**	1.42	15.73
Transverse	No support v Lower Lumbar	−3.72	−8.5	0.129	−8.75	1.32
	No support v Lower Thoracic	−7.23	−16.6	**0.038**	−13.97	−0.49
	No support v Upper Thoracic	7.11	16.3	**0.040**	0.40	13.82
	Lower Lumbar v Lower Thoracic	−3.52	−7.4	0.240	−9.84	2.81
	Lower Lumbar v Upper Thoracic	10.83	22.8	**0.011**	3.15	18.50
	Lower Thoracic v Upper Thoracic	14.34	28.2	**0.015**	3.53	25.16
Sagittal	No Strap v Strap	−12.00	−17.5	**0.034**	−22.86	−1.15
Transverse	No Strap v Strap	−8.95	22.3	**0.018**	−15.96	−1.95
Coronal	Left v Right	5.28	14.6	**0.017**	1.18	9.39

## Data Availability

Data supporting the results of this study are available from the corresponding author upon reasonable request.
